# Dry Needling Related Short-Term Vasodilation in Chronic Sciatica under Infrared Thermovision

**DOI:** 10.1155/2015/214374

**Published:** 2015-03-02

**Authors:** Elżbieta Skorupska, Michał Rychlik, Wiktoria Pawelec, Włodzimierz Samborski

**Affiliations:** ^1^Department of Rheumatology and Rehabilitation, Poznan University of Medical Sciences, Fredry 10, 61-701 Poznan, Poland; ^2^Department of Virtual Engineering, Poznan University of Technology, Plac Marii Skłodowskiej-Curie 5, 60-965 Poznan, Poland; ^3^Department of Biomechanics, University School of Physical Education, Królowej Jadwigi 27/39, 61-871 Poznan, Poland

## Abstract

Vasomotor responses to dry needling (DN) of trigger points (TrPs) under infrared thermovision (IRT) camera control and TrPs coexistence in chronic sciatica patients have never been studied. *Materials and Methods*. Fifty consecutive chronic sciatica patients were enrolled in the study. DN under IRT control was performed for all patients regardless of gluteus minimus (GM) active TrPs examination. Then, the vasomotor response and its agreement with TrPs examination were evaluated. *Results*. The prevalence of GM active TrPs was 32%. DN provokes intensive vasodilatation for TrPs-positive patients only, with the localization dependent on referred pain during the procedure (*r* = 0.896;  *P* = 0.000) not the daily complaint. The increase of vasodilatation was, for example, for thigh, TrPs-positive +30.29% (*P* < 0.05) versus TrPs-negative +4.08%. Additionally, a significant skin temperature increase was observed for TrPs-positive only, for example, thigh +1.5 ± 1.3°C (maximum) and +1.2 ± 1.0°C (average) (both *P* < 0.05). *Conclusion*. GM active TrPs prevalence among chronic sciatica patients was around one in three. Every TrPs-positive subject presented with vasodilatation under IRT in the area of DN related referred pain. Although TrPs involvement in chronic sciatica patients is possible, further studies on a bigger group of patients are still required.

## 1. Introduction

Dry needling (DN), called western acupuncture, is a common treatment technique for myofascial pain syndrome (MPS). MPS is caused by the presence of trigger points (TrPs) which are defined as hyperirritable nodules within taut bands of skeletal muscles and are divided into active and latent [1–3]. An active TrP is characterized by spontaneous pain or pain in response to movement, stretch, or compression, while a latent TrP is a sensitive spot with pain or discomfort in response to compression only [1]. Currently, diagnosis of MPS is based on the palpatory clinical criteria defined by Travell and Simons [1]. Although identification of TrPs is clinically difficult, recent research provides strong evidence that TrPs exist [4–9]. The lack of objective diagnostic criteria provokes a lot of controversy regarding their diagnosis. One of the most important confirmatory signs of TrPs diagnosis is recognition of referred pain, which occurs when spontaneous pain is referred to sites remote from TrPs [1, 10]. Dry needling intensifies referred pain when the needle is inserted [2, 3] and it was lastly presented using infrared thermovision (IRT) camera control that DN related short-term vasodilation occurred in a chronic sciatic leg pain [11, 12]. Moreover, some meaning of IRT observation of TrPs related referred pain has been demonstrated before [13, 14]. The presence of autonomic phenomena in the area of TrPs referred pain has been postulated for years but limited to severe active TrPs only [1]. The vasomotor reactions are one of the possibilities. It is widely accepted that skin temperature (*T*_sk_) changes in biological systems are defined as the difference of thermal energies received and lost by conduction transfer and advection, as well as metabolic reactions, for example, chemically released ones, and can be observed by IRT [15, 16]. If the presence of vasomotor phenomena in the referred pain area was confirmed for every TrP, then DN under IRT control could be a promising direction in the objective confirmation of TrPs diagnosis.

However, the presence of autonomic phenomena, for example, vasoconstriction and vasodilation, coincident with TrP referred pain area was thought to be limited to severe active TrPs exclusively [1]. It is not known whether every active TrP can develop DN related vasodilation. Additionally, it is not known how often TrPs coexist in sciatica. However, in more recent studies, the involvement of the autonomic nervous system (ANS) in muscle pain at active and latent trigger points has been suggested [17–21]. Moreover, the involvement of ANS in pain propagation in around 30% of sciatica patients has been suggested [22] and as it is known the prevalence of TrPs reaches approximately 30–50%, especially among patients in chronic stage [1]. All of these data indicate that the prevalence of TrPs in sciatica, especially chronic, can be considerable.

The aim of this study was to (1) evaluate the prevalence of active gluteus minimus trigger points among chronic sciatica patients; (2) assess vasomotor responses to dry needling under IRT control of TrPs-positive and TrPs-negative sciatica patients and their agreement with the results of palpatory TrPs diagnostic criteria by Travell and Simons [1].

## 2. Materials and Methods

### 2.1. Ethics Statement

The study was conducted in accordance with the Declaration of Helsinki approved by the Ethics Committee of Poznan University of Medical Sciences (number 630/13). All subjects gave written informed consent to participate in the study before data collection. A detailed description of all examination and treatment procedures, including dry needling and risks involved in the study, was provided to the participants. The participants had the right to refuse DN treatment and withdraw from the study at any time without penalty.

### 2.2. Subjects

Fifty consecutive patients with chronic sciatica from the University Pain Clinic (21 men and 29 women; mean age 47.50 ± 10.82 y; pain level on Visual Analogue Scale (VAS) 6.13 ± 2.47; symptoms duration 5 ± 1.2 months) were enrolled in the experimental study. All participants were diagnosed by an experienced neurologist based on magnetic resonance imaging evaluation and clinical bedside examination accompanied by a positive straight leg test. Participants were to manifest negligible pain symptoms in the contralateral leg. Key inclusion criteria were as follows: age between 30 and 60 (inclusive), both lower limbs present, and history of unilateral low back pain radiating down to lower extremities (a minimum of 3 months) with leg pain ≥ 3 on 1–10-point scale VAS, with this being the dominant pain problem. Patients were excluded from a study for a number of reasons, that is, Cauda Equina Syndrome, previous back surgery, spinal tumors, pregnancy, coagulant treatment, disseminated intravascular coagulation, diabetes, epilepsy, infection, inflammatory rheumatologic diseases, stroke, and oncological history.

### 2.3. Methods

#### 2.3.1. Myofascial Pain Diagnosis

All subjects were evaluated towards myofascial pain syndrome coexistence based on Travell and Simons diagnostic criteria, namely, (1) taut band palpable (if muscle accessible), (2) exquisite spot tenderness of a nodule in the taut band, (3) patient's recognition of current pain complaint by pressure on the tender nodule (identifies an active trigger point), and (4) painful limit to full stretch range of motion [1]. The taut band of the gluteus minimus muscle is unlikely to be palpated because it lies deeper than both the gluteus maximus and the gluteus medius muscle. However, TrP spot tenderness can be clearly localized. Among the subjects enrolled in the study, the presence of active trigger points in the gluteus minimus muscle was diagnosed when 2–4 criteria were confirmed and digital pressure of tender spot provoked referred pain typical of gluteus minimus trigger points familiar to the patients, namely, TrPs in the anterior portion of muscle fibers referred pain and tenderness to the lower lateral part of the buttock, the lateral aspect of the thigh and knee, and to the peroneal region of the leg as far as the ankle. In the case of TrPs in the posterior portion of muscle fibers, pain was referred to the lower lateral part of the buttock down to the lateral and posterior aspect of the thigh and calf [1]. For the subjects examined as TrPs-negative, the two most tender spots within the gluteus minimus were chosen.

#### 2.3.2. The Procedure of Thermovision Camera Control

*(A) The IRT Statement*. A thermovision touchless camera (NEC-AVIO TVS-200EX) using a 8–14 *μ*m wave band, temperature resolution better than 0.08°C, and sensitivity 80 mK and working in real time was applied. The camera was equipped with a high-speed (60 Hz) uncooled FPA 320 × 240 (*H* × *V*) pixels VOx (vanadium oxide) microbolometer. The equipment allows taking images with spatial resolution of 1.68 mrad and field of view (FOV) 30.6° × 23.1° (*H* × *V*). For data analysis (thermal images), a specialist program “Thermography Studio 2007 Professional” was used.

Thermographic images were recorded by an expert following a standard protocol recommended by the Academy of Neuro-Muscular Thermography. The expert also evaluated the images [23]. Patients were instructed to avoid physiotherapy and manual therapy 24 hours prior to the test and to avoid using nasal decongestants, analgesics, anti-inflammatory drugs, or any substances affecting the sympathetic function. They were also instructed not to drink coffee or alcohol and refrain from smoking 2 hours before the recording. For each examination of the patient, the room temperature was measured with an electronic thermometer equipped with a hygrometer (humidity). The measurement results of both values (temperature and humidity) were taken into consideration (thermal image correction) in the analysis of the thermographic images. In addition, the thermal camera was equipped with an internal ambient temperature sensor. The average value of room temperature ranged from 24 to 26 degrees Celsius and humidity was about 45–55%. During examinations, the patients were half nude (from the waist down)—only light top shirts were permitted.

To obtain the stability of patient body temperature and to ensure the adjustment of the recording camera's temperature to the interior conditions, the evaluation began 30 minutes after the patient had entered the examination room. Thermal isolation of the evaluated area from other thermal factors that might have influenced the evaluation, including other parts of the patient's and doctor's bodies, was ensured. Moreover, when performing thermovision imaging, the general rules of camera usage were followed.

*(B) Dry Needling*. The patient's position for needling of the gluteus minimus muscle was side-lying position. The muscle was needled with flat palpation perpendicular to the muscle along the counter of the iliac crest. Strong depression of the subcutaneous tissue was applied in order to reduce the distance of the skin from the muscle. Depth of penetration depended on the amount of adipose tissue [3]. Therapeutic needling was performed with 0.30 mm diameter, 60 mm long sterile acupuncture needles SE L (Serin Corp, Shizuoka, Japan). Each needle was packed separately.

*(C) The Protocol of Dry Needling under Infrared Thermography Control*

*Phase I (Patient's Preparation)*. The patients were asked to draw their current pain pattern on the diagram. Once the drawing was complete, based on the diagnostic criteria set by Travell and Simons, trigger points coexistence was confirmed [1]. In the examined muscle, two points for needle insertion were marked. While active trigger points were marked in the case of TrPs-positive subjects, for TrPs-negative subjects two most tender spots were selected.

*Phase II (Dry Needling under IRT Control)*

- Part A: IRT side-to-side comparison of the painful subarea to the same subarea in the opposite extremity of the patient at rest (standing position).
- Part B: dry needling session under IRT control.

For part B, the area to be observed by IRT was chosen according to the gluteus minimus referred pain pattern. The examined patients were positioned on the side, on the uninvolved extremity with the hip and knee flexed. In this position, thermovision images of the patient were recorded.

For adequate representation of dimensions, a calibration standard has been applied. The next step involved recording the “base” image. The image was recorded to establish the initial level of the patient's temperature parameters. When all of the above-mentioned conditions were met, needling of two previously marked points was performed.

The needle pierced the skin and reached the painful point with referred pain. The recognizable pain started to withdraw partially and repeatedly and eventually both the pain and muscle fiber contraction subsided. The time of needling was 5 minutes for any given point.

During the whole procedure, the subarea of referred pain reported by the patient (thigh, calf, and foot) was recorded. At the end of the procedure, the patients were asked to answer the question “Was the pain evoked by needling similar to your daily pain?”

*Phase III (Post-DN Observation under IRT Control)*. After the needling of both marked points was ended, further thermovision imaging was performed. The IRT observation lasted for six consecutive minutes after DN.

*Phase IV (Infrared Thermovision Camera Data Analysis)*. The thermovision material was gathered during the evaluation stage of the study. The study consisted of the following:

- side-to-side comparison (the comparison of data concerning the average surface temperature of the painful area and the analogous area in the other (pain-free) part of the body): before using the technique of dry needling, each patient was subject to infrared observation of the initial state. In each case, four infrared images on each side of the patient (front, back, left, and right side) and one additional image of the foot (top view) were taken. To avoid the effect of the shortcut of the image, the thermovision camera was placed at the knee height. The particular regions of interest—thigh, calf, and foot—were compared, respectively, with each other (region of the left and right leg);
- comparative assessment of thermovision material representing the following stages of dry needling under ITR control:
  - state before procedure,
  - state directly after DN (phase II),
  - observation of the subject for six consecutive minutes after DN (phase III).

According to the literature, autonomic phenomena of TrPs can occur in the referred pain area. TrPs of the gluteus minimus muscle can refer pain to the whole lower limb (buttock, thigh, calf, and rarely foot) or one part of the lower limb, for example, buttock and thigh only. Because of that the evaluated leg was divided into three measurement subareas (thigh, calf, and foot). Within every subarea, the measurement of the following parameters was recorded:

- temperature value of the highest temperature point in a given subarea,
- maximum, minimum, and average temperature value of a given subarea,
- values of the surface areas (isothermal-areas measured in cm^2^) of constant temperature.

*(D) Thermogram Analysis towards Vasomotor Reactions Presence*. After the infrared image calibration procedure, the measurements of area size were performed automatically in Thermography Studio 2007 Professional. As a result of scaling, each of the image pixels obtained size in mm^2^ corresponding to the actual size of the area. To scale the size of objects recorded on thermographic images, the pattern (standard) length was used. After placing the patient in the position for the examination, a special “reference” image using the standard length was performed. Then, before the analysis was conducted, each series of thermal images performed for the patient was calibrated using the calibration element registered in the image. The calibration pattern was placed in the immediate vicinity of the patient's feet. In the software (Thermography Studio), the calibration element was defined by corresponding length and in this manner thermographic images were scaled to the real size.

On each recorded thermogram, in the whole measurement area (thigh, calf, and foot), 0.7°C isothermal-areas were identified (range of temperature from 23.7 to 40.0°C). According to the three-sigma rule, data were grouped for three temperature ranges (low range 28.4–29.2°C, middle range 29.9–32.8°C, and high range above 33.6°C). The calculations were performed for particular subareas (thigh, calf, and foot). The data obtained for three range temperature areas in cm^2^ were calculated to percentage.

If the high range temperature increase was observed, the isothermal-areas higher than *T*_max⁡_ before procedure were isolated (gray picture).

### 2.4. Statistical Analysis

Exact one- and two-way Mann-Whitney *U* tests were performed in order to observe that data are representative of the full population of possible data values. Tests were applied to compare the differences in the three temperature ranges (low, middle, and high range temperature) for average temperature (*T*_avr_), maximum temperature (*T*_max⁡_), and percentage size of isothermal-areas (low, middle, and high).

Pearson correlation with two-tailed significance test was applied to define the dependency of vasomotor phenomenon occurrence. All comparisons were completed, with trigger points coexistence being the differentiating criterion. Values in text, figures, and are expressed as ± standard error of the mean (SEs). Significance level was set at *P* < 0.05. Statistical analysis was performed using IBM SPSS Statistics, version 20.

## 3. Results

In the group of subjects, the prevalence of gluteus minimus active trigger points (TrPs) was 32%. All TrPs-positive subjects recognized referred pain evoked during dry needling (DN) as their daily pain. Side-to-side (painful to nonpainful leg) average skin temperature (*T*_avr_) comparison before dry needling session revealed Δ*T*_avr_ of 0.01°C. DN related referred pain was reported during the procedure by every TrPs-positive subject in the thigh and by every second TrPs-positive subject in the calf. Among non-TrPs subjects some patients (*n* = 5) were needle reactive and reported needle sensation on the thigh.

**Figure 1:**
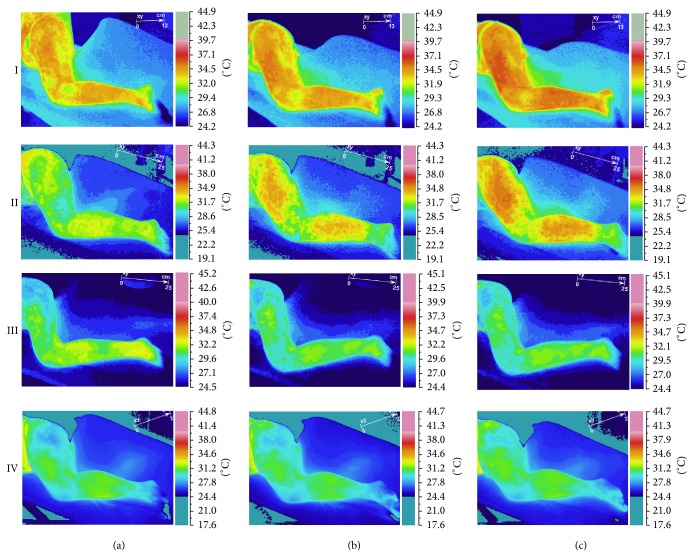
Temperature reaction measured on skin surface (short-term vasodilation effect). In rows, cases one and two (TrPs-positive) and three and four (non-TrPs) were shown. In columns, (a) state before DN (initial state), (b) state immediately after DN (phase II), and (c) state immediately after observation (phase III) were presented.

**Figure 2:**
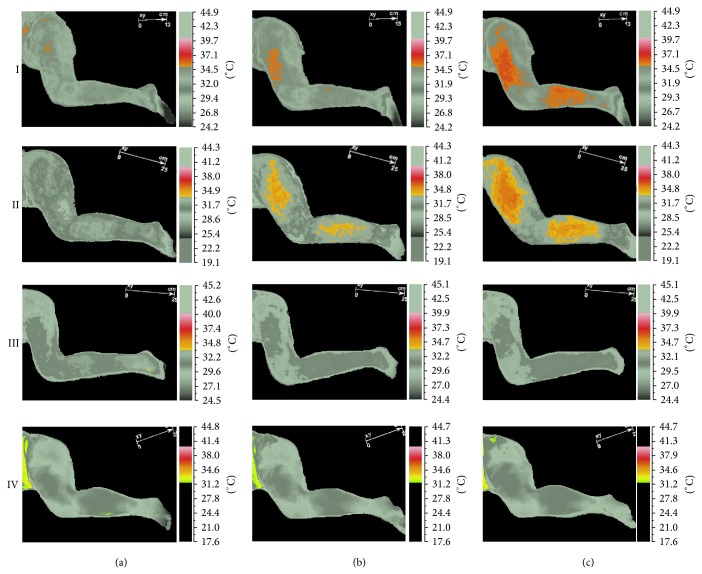
Visualization of short-term vasodilation (the area of vasodilation higher than *T*_max⁡_ of the initial state). In order to describe the area of short-term vasodilation using IRT procedure, patient temperature reaction was isolated—grey pictures column. In rows, cases one and two (TrPs-positive) and three and four (non-TrPs) were shown. In column (a) pre-DN (initial) state, in column (b) vasomotor referred pain after DN (phase II), and in column (c) the vasomotor referred pain after observation (phase III) were presented.

Infrared thermovisual camera revealed DN-related short-term vasodilation in the area of expected vasomotor reactions (thigh and calf) (Figure 1). The presence of the vasodilatation higher than *T*_max⁡_ before procedure was confirmed for TrPs-positive subjects exclusively (Figure 2). Pearson correlation with two-tailed significance test confirmed that the vasodilatation was dependent on TrPs codiagnosis and recognition of gluteus minimus referred pain evoked during dry needling-IRT session (*r* = 0.896, *P* < 0.005). Detailed analysis of measured subareas confirmed the same (thigh *r* = 0.896, *P* < 0.005; calf *r* = 0.606, *P* < 0.005).

The exact two-way Mann-Whitney* U* test confirmed that dry needling provoked a significant percentage high temperature (HT%) isothermal-area increase only when TrPs coexisted (phases II and III both *P* < 0.05). That HT% increase was significant in the area of the expected vasomotor reactions (thigh and calf; both *P* < 0.05; exact two-way Mann-Whitney* U* test). For the foot, an insignificant decrease of HT% was confirmed. Among TrPs-negative patients, an insignificant HT% decrease (calf and foot) and an insignificant HT% increase (thigh) were observed.

**Figure 3:**
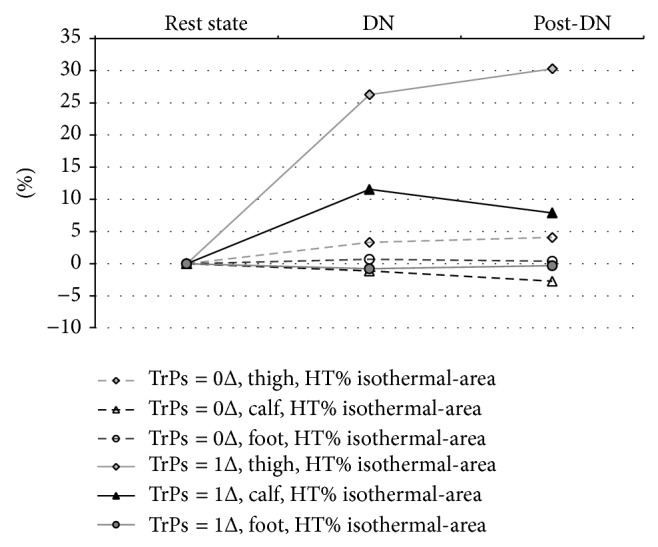
Changes of Δ isothermal-area percentage for high range temperatures using IRT procedure. In the group, short-term vasodilation of the thigh and calf is related to TrPs codiagnosis. The reaction is characteristic of the thigh in all cases and for some subjects also of the calf. The reaction for the foot was similar for both TrPs-positive and non-TrPs subjects. Among non-TrPs subjects, a small insignificant increase/decrease of isothermal-areas was observed. Differences of HT% isothermal-area depending on IRT procedure phases were as follows. TrPs-positive subjects: (a) thigh phase II 26.27%; phase III 30.29%; (b) calf phase II 11.56%; phase III 7.91%; (c) foot phase II −0.77%; phase III −0.32%. TrPs-negative subjects: (a) thigh phase II 3.30%; phase III 4.08%; (b) calf phase II −1.13%; phase III −2.73%; (c) foot phase II 0.68%; phase III 0.37%.

Detailed values of HT% changes dependent on TrPs presence were shown in Figure 3.

**Figure 4:**
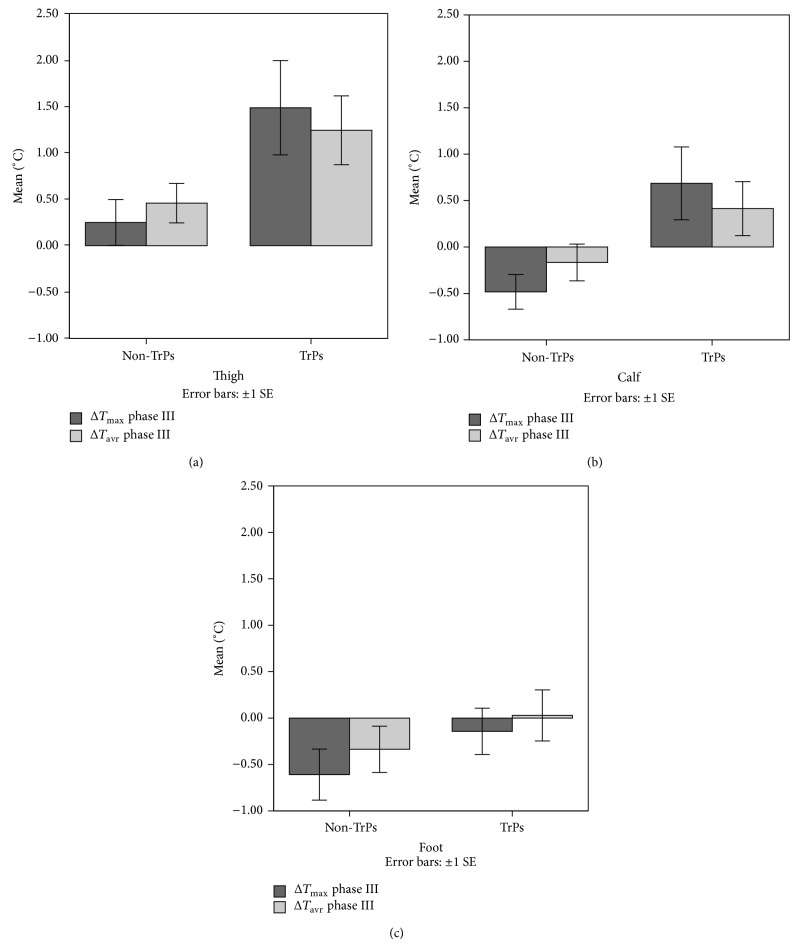
Skin temperature differences depending on TrPs codiagnosis and IRT procedure. DN provoked a statistically significant increase of *T*_sk_ (*T*_max⁡_, *T*_avr_) (both *P* < 0.05) in the thigh and calf in the area of the expected vasomotor reactions (TrPs-positive subjects). On the contrary, among TrPs-negative subjects, a *T*_sk_ decrease in the calf and foot was confirmed. However, an insignificant thigh *T*_sk_ increase was present (without vasodilatation on a gray thermogram).

The exact two-way Mann-Whitney* U* test confirmed that dry needling provokes a significant skin temperature (*T*_sk_) increase (maximum and average) for TrPs-positive patients only for both phases (namely, II and III; both *P* < 0.05). Additionally, the significant *T*_sk_ increase was confirmed only in the area of the expected vasomotor reactions (thigh and calf, both *P* < 0.05; exact two-way Mann-Whitney* U*) (Figure 4). On the contrary, among non-TrPs subjects, a *T*_sk_ decrease in the calf and foot and insignificant thigh *T*_sk_ increase without vasodilatation were observed on the gray thermogram (Figure 4).

Among TrPs-positive subjects, the value of a significant maximum temperature increase in phases II and III, respectively, was for the thigh +1.29 ± 1.22°C and +1.5 ± 1.30°C; for the calf +0.46 ± 0.71°C and +0.7 ± 1.0°C. The value of a significant average temperature increase in phases II and III, respectively, was for the thigh 1.31 ± 0.88°C and 1.2 ± 1.0°C; for the calf 0.54 ± 0.72°C and 0.4 ± 0.8°C.

## 4. Discussion

This study demonstrated that at least one in three chronic sciatica patients presented with gluteus minimus TrPs coexistence. Dry needling under IRT control of sciatica subjects revealed vasodilation coincident with the patient's pain area only when TrPs coexisted (Figures 1 and 2). This is the first study that records DN related vasodilation in the area of TrPs referred pain, which supports the theory that in the area of a TrP referred pain some vasomotor reactions can happen [24]. Moreover, the fact that every TrPs-positive subject presented with vasodilatation denies the opinion that the presence of autonomic phenomena, for example, vasomotor reactions in the area of referred pain, is limited to severe active TrPs exclusively. The occurrence of vasodilation in the present study was dependent on TrPs coexistence and referred pain reported during the procedure (thigh *r* = 0.896, *P* < 0.005; calf *r* = 0.606, *P* < 0.005) not the daily pain reported by the patients (Figure 2). Additionally, the presence of vasodilatation provoked a significant *T*_sk_ increase (maximum, average both *P* < 0.05) for the thigh and calf (Figure 4). Although no study of *T*_sk_ reactions after DN has been conducted so far, Zhang et al. found no *T*_sk_ reactions after noxious stimulation of latent TrPs [21]. Interestingly, contrary to this study, Kimura et al. found a significant decrease in *T*_sk_ (*P* < 0.05) after glutamate injection to latent TrPs and no reactions after injection to non-TrPs [20]. It is difficult to explain the contrary results at this moment. Hypothetically, it is possible that the results of this study were caused by including subjects who were sciatica patients with active TrPs and not healthy subjects with latent TrPs. Additionally, maybe not only the type of TrPs (active or latent) but also the form of the main disease (acute or chronic) has some meaning. Apart from that, different types of examined muscles (the region of the body) can probably present different *T*_sk_ reactions. All of these doubts should be looked at in the future. Another hypothetical explanation can be given by Sandberg et al. [25]. They proved that the value of skin and muscle blood flow increase depends on needling intensity [25]. The highest values are obtained after moving the needle inserted to the muscle acupoint as compared with inserting the needle only into the skin or deeper into a muscle. The acupuncture technique of needling demands inserting the needle into the muscle with/without moving it in circles, but dry needling is much more aggressive and requires repetitive movements of the needle up and down so as to evoke twitch response. Nevertheless, acupuncture studies indicate that after needle or manual stimulation of acupoints the vasodilation is only local, around 5–10 cm from the needling point, or the lack of vasomotor reaction is observed [25, 26]. Surprisingly, in the current study, a far wider vasodilation spread from needling point has been observed (Figures 1 and 2).

On the other hand, it may not be the kind of noxious stimuli per se but rather the time of stimulation which explains such intensive vasomotor reactions. Additionally, evoking the twitch response, which is a spinal cord reflex characteristic of TrPs only, might have some meaning for revealing vasodilatation. Further studies are required to resolve these doubts.

However, acupuncture studies show that needle stimulation of acupoints provokes both decrease and increase in *T*_sk_ of around ±1.0°C [25, 27]. There is no available IRT study on pain patients after noxious stimuli. The only one (low back pain patients after cold exposure) revealed regional Δ*T*_sk_ of more than 0.3°C, with vessel reactions occurring mainly distally (the feet) [28]. The outcome of Δ*T*_sk_ in the present study is much higher than the one achieved by cold exposure but it is similar or just a little higher when compared with outcomes of acupuncture needle stimulation. However, acupuncture stimulation provoked vasodilation only locally.

What distinguished this study is not only the presence of short-term vasodilation but also its size and localization. All of the TrPs-positive patients reported vasodilatation far away from the needling point (thigh and calf), coincident with the area of the expected TrPs referred pain. However, a significant HT% increase (*P* < 0.05) was confirmed only for the thigh. According to the theory of myofascial pain syndrome, TrPs referred pain may follow its pattern either in full or in part; thus the same is observed in the present study. Only half of the TrPs-positive patients reported DN sensation in the calf. Moreover, significant widening of thigh HT% isothermal-area in TrPs-positive patients is contrary to an insignificant slight widening or shrink in non-TrPs subjects (Figure 3). Interestingly, some of the non-TrPs patients reported thigh needle sensation, which can explain the little thigh HT% increase (Figure 3). Nevertheless, there was no vasodilatation on the gray picture apart from the hot spots for TrPs-negative patients. However, this needle reaction of TrPs-negative subjects is interesting in the light of gluteus minimus TrPs theory [1]. TrPs of the gluteus minimus muscle lie deeply and the presence of referred pain (one of the most important confirmatory signs) can be confirmed by needle, not the snapping palpation [1]. It is possible that needle reactive non-TrPs subjects might have had a latent form or mild active forms of TrPs, which are not able to provoke vasodilatation on the gray picture (Figure 2) but HT% increase is visible. To answer these questions, further studies are required.

### 4.1. Hypothetical Biological Explanation of Short-Term Vasodilatation Presence

It is known that, in nonglabrous or hairy areas of skin (limbs, head, and trunk), reflex changes in skin blood flow (SkBF) are mediated by two branches of the sympathetic nervous system (noradrenergic vasoconstrictor nerves and cholinergic active vasodilator nerves, which are unique to humans). Despite the fact that the cutaneous active vasodilator system is the most studied one, especially SkBF, the precise mechanisms thanks to which the cutaneous active vasodilator system functions remain somewhat enigmatic [29]. In this study, the presence of vasodilatation was related to mechanical stimulus (dry needling). Generally, the mechanisms underlying the translation of mechanical stimuli into biochemical information have a fundamental function in physiology and pathophysiology but are only poorly understood [30, 31]. Similarly, the exact mechanism of DN remains unexplained. Some authors propose that needling of active TrPs can stimulate sensory afferent A*δ* nerve fibers or C-nerve fibers [2]. It is known that noxious stimulation of C-fiber terminals can release vasoactive substances such as substance P and calcitonin gene-related peptide (CGRP). Additionally, these vasoactive neuropeptides produce a flare that develops around the primary activation site via axon reflex vasodilatation, which is observed, for example, after needle stimulation of acupoints [26, 32–34]. Moreover, when these neuropeptides remain below the critical level at flare borders, the flare size shrinks [35]. In this study, the widening of vasodilation was observed, and then the vessel reactions shrank.

Additionally, it seems that the biphasic nature of vasodilatation (Figure 3) is similar to that described by Kellogg Jr. et al. [29], who observed biphasic vasodilation because of local warming with an initial increase mediated by an axon reflex followed by a plateau phase which requires nitric oxide generation by nitric oxide synthases. Unfortunately, the nature of vasodilatory neurotransmitter of the initial phase of local vasodilation remains uncertain [29]. Moreover, while previous studies suggested that the axon reflex contributes to cutaneous vasodilation in response to acupuncture stimulation, one of the latest acupuncture studies suggested that the nitric oxide mechanism is responsible for it [36]. No matter which neurotransmitters exactly are involved, it provides an explanation for local vasodilatation, not so extended and coincident with DN related referred pain vasodilatation spread to the thigh and calf. Available experimental evidence suggests that manoeuvres increasing sympathetic outflow can enhance motor unit activity and facilitate muscle pain at TrPs [17, 18, 37]. The involvement of antidromic axon reflexes resulting from SkBF after noxious stimulation of latent TrPs was suggested [21]. Additional hypothetical explanation for presence of TrPs related vasodilatation can be given thanks to the work of Hong who described “TrPs circuits,” that is, a neural network of “TrPs related sensory nerves” which can explain the widening of vasodilation more than several centimeters from needling point. This neural network is organized by the connection between nociceptors in a TrP region and some group of dorsal horn cells (sensory neurons) in the spinal cord [38–40]. Hong is convinced that “TrPs circuits” are responsible for all TrP phenomena including pain, referred pain, local twitch response, motor dysfunction, and autonomic phenomena via the spinal cord reflex [40, 41]. His theory seems plausible and it is possible that DN of active TrPs can probably excite “TrPs circuits.”

### 4.2. Study Limitations

This study is limited by the absence of a control group. DN provoked significant vasodilation above the initial maximal temperature before procedure for TrPs-positive subjects. This was not observed for TrPs-negative subjects. Thus, it is required that the influence of DN on healthy subjects is examined. Another limitation of the study is the lack of the analysis of the TrPs-negative subjects who reported needle sensation as likely TrPs. According to the literature, it is more probable to evoke the referred pain from TrPs by needle than by snapping palpation [1]. Due to this fact, the results obtained for TrPs-negative patients might have inadequately reflected the changes recorded by IRT in that subgroup. Further studies, for example, crossover study, on dry needling under IRT control are required.

## 5. Conclusion

The prevalence of active trigger points among chronic sciatica patients was around one in three. Every TrPs-positive subject presented with dry needling related short-term vasodilatation in the area of referred pain. Although the involvement of TrPs in chronic sciatica patients is possible, further studies on a bigger group of patients are still required.

## Abbreviations

ANS: Autonomic nervous system
HT% isothermal-area: High temperature isothermal-area
DN: Dry needling
IRT: Infrared thermovision camera
MPS: Myofascial pain syndrome
MT% isothermal-area: Middle range temperature isothermal-area
*T*_avr_: Average temperature
*T*_max⁡_: Maximum temperature
TrPs: Trigger points
*T*_sk_: Skin temperature
VAS: Visual Analogue Scale
Δ*T*: Temperature difference.
